# Structural brain changes associated with antipsychotic treatment in schizophrenia as revealed by voxel-based morphometric MRI: an activation likelihood estimation meta-analysis

**DOI:** 10.1186/1471-244X-13-342

**Published:** 2013-12-20

**Authors:** Ulysses S Torres, Eduardo Portela-Oliveira, Stefan Borgwardt, Geraldo F Busatto

**Affiliations:** 1 Post-Graduate Program in Radiology, Institute of Radiology (INRAD), University of Sao Paulo Medical School, Sao Paulo, Brazil; 2 Laboratory of Neuroimaging in Psychiatry (LIM-21), Institute of Psychiatry, University of Sao Paulo Medical School, Centro de Medicina Nuclear, 3º andar, Rua Dr. Ovídio Pires Campos, s/n, Sao Paulo, Sao Paulo, 05403-010, Brazil; 3 Center for Interdisciplinary Research on Applied Neurosciences (NAPNA), University of São Paulo, Sao Paulo, Brazil; 4 Department of Radiology, Hospital de Base, São José do Rio Preto Medical School, Sao Paulo, Brazil; 5 Department of Psychiatry, University of Basel, Basel, Switzerland; 6 Department of Psychosis Studies, Institute of Psychiatry, King’s College, London, UK

**Keywords:** Schizophrenia, Antipsychotics, Voxel-based morphometry, Magnetic resonance imaging, Neuroimaging

## Abstract

**Background:**

The results of multiple studies on the association between antipsychotic use and structural brain changes in schizophrenia have been assessed only in qualitative literature reviews to date. We aimed to perform a meta-analysis of voxel-based morphometry (VBM) studies on this association to quantitatively synthesize the findings of these studies.

**Methods:**

A systematic computerized literature search was carried out through MEDLINE/PubMed, EMBASE, ISI Web of Science, SCOPUS and PsycINFO databases aiming to identify all VBM studies addressing this question and meeting predetermined inclusion criteria. All studies reporting coordinates representing foci of structural brain changes associated with antipsychotic use were meta-analyzed by using the activation likelihood estimation technique, currently the most sophisticated and best-validated tool for voxel-wise meta-analysis of neuroimaging studies.

**Results:**

Ten studies (five cross-sectional and five longitudinal) met the inclusion criteria and comprised a total of 548 individuals (298 patients on antipsychotic drugs and 250 controls). Depending on the methodologies of the selected studies, the control groups included healthy subjects, drug-free patients, or the same patients evaluated repeatedly in longitudinal comparisons (i.e., serving as their own controls). A total of 102 foci associated with structural alterations were retrieved. The meta-analysis revealed seven clusters of areas with consistent structural brain changes in patients on antipsychotics compared to controls. The seven clusters included four areas of relative volumetric decrease in the left lateral temporal cortex [Brodmann area (BA) 20], left inferior frontal gyrus (BA 44), superior frontal gyrus extending to the left middle frontal gyrus (BA 6), and right rectal gyrus (BA 11), and three areas of relative volumetric increase in the left dorsal anterior cingulate cortex (BA 24), left ventral anterior cingulate cortex (BA 24) and right putamen.

**Conclusions:**

Our results identify the specific brain regions where possible associations between antipsychotic drug usage and structural brain changes in schizophrenia patients are more consistently reported. Additional longitudinal VBM studies including larger and more homogeneous samples of schizophrenia patients may be needed to further disentangle such alterations from those possibly linked to the intrinsic pathological progressive process in schizophrenia.

## Background

Schizophrenia is a common, complex and severe psychiatric disorder affecting approximately 1% of the world population. The disorder remains a major cause of chronic disability among young and working-age individuals and is associated with a significant health, social and economic burden internationally [1-4]. Whereas finding an etiology for schizophrenia has been considered the “Holy Grail” of biological psychiatry research for more than one hundred years [5,6], its neurobiological basis mostly remains elusive in terms of its major neuropathologic, pathophysiologic, psychopharmacologic and genetic aspects [1,7].

In the last decades, with the advent of more sophisticated neuroimaging techniques such as magnetic resonance imaging (MRI), which allows *in vivo* studies of the brains of individuals with schizophrenia, structural brain changes in schizophrenia have been extensively characterized [8-10]. Some of these findings include smaller mean cerebral volumes and greater mean total ventricular volume in patients with schizophrenia, with significant decreases in both gray and white matter [11].

These findings initially have favored a dominant “neurodevelopmental” model of the origin of the disease: in the model, schizophrenia is basically a consequence of a disruption in early brain development, long before the clinical manifestations of disease that typically occur in adolescence or early adulthood. Moreover, an interaction between these early brain insults and environmental factors delineating the brain maturation in adolescence would be necessary to trigger psychotic behavior [7,12-17].

However, as these structural brain changes are often subtle and their course is difficult to appreciate in an evolving manner, it is only after robust and longitudinal MRI studies that the possibility of progressive structural brain changes over time has been strengthened (favoring the addition of a “neurodegenerative” hypothesis to the dominant “neurodevelopmental” model) [18-25]. The advent of voxel-based morphometry (VBM) was of crucial importance in this sense, as VBM represents an automated method of measuring whole-brain morphometry by comparing groups of images on the relative local concentration or density of gray or white matter in a voxel-by-voxel way, thus reducing investigator bias and providing highly reproducible results, among other benefits [26-28].

Although a neurodevelopmental insult does not preclude an associated neurodegenerative process [29], the idea of progressive structural changes in the brain over time, which could denote neurodegeneration, has been a controversial issue [30-32], particularly because the findings of different studies have at times seemed inconsistent. A notable example is the question of lateral ventricles: whereas some longitudinal MRI and CT studies showed no enlargement over time [33-35], others have shown significant progression [36]; a recent meta-analysis comprising 13 studies regarding this question identified evidence of a progressive ventricular enlargement, concluding that the exclusively neurodevelopmental model of schizophrenia is now challenged [30].

While the progressive nature of structural brain changes in schizophrenia is not yet fully understood, they are thought to be in some degree due to a combination of abnormalities of synaptic plasticity, abnormal brain maturation and distinct environmental factors [37]. For many years, although a significant number of individuals involved in neuroimaging studies had used antipsychotic drugs, the role of drug treatment as a cause of these changes has been scarcely investigated [38]. Thus, among the environmental factors, a major current question is the role of antipsychotic medications in the progression of structural abnormalities, i.e., to determine to what extent these global brain volume changes are uniquely a consequence of schizophrenia (a progressive pathophysiology of the illness) or an effect of antipsychotics (including the potentially different role of typical and atypical classes) [38-43].

Antipsychotic medications are the cornerstone of the treatment of schizophrenia and have a positive effect on the prognosis, not only by leading to a general improvement in the long-term outcomes of patients but also by reducing the severity and frequency of positive and negative psychotic symptoms, including suicide risk as well as behavioral disturbances [44-49]. Classical (typical) antipsychotics (e.g., haloperidol) predominantly act by blocking dopamine D_2_ receptors in mesostriatal, mesolimbic and mesocortical regions and the thalamus, directly contributing to the amelioration of psychotic symptoms that are thought to be a result of abnormally increased dopaminergic activity in these pathways [50-53]. New-generation (atypical) antipsychotics (e.g., clozapine), despite their activity in reducing dopaminergic activity through dopamine D2 receptors blockade, have binding activity at various others receptors, including a higher affinity for the serotoninergic 5-HT_2A_receptors (high 5-T_2A_/D_2_ binding ratio) involved in the treatment of positive and negative symptoms [53,54]. Although this differentiation has clinical relevance, especially with regard to distinct side-effect patterns, the mechanisms of action of these drugs are not yet fully understood, and the definition of atypicality remains a matter of discussion [53-55].

Neuroimaging has a potential role in research aimed at a better understanding of structural brain changes secondary to antipsychotic usage. In the last years, numerous studies have been undertaken to address these questions, involving variable subsets of schizophrenia patients with different disease duration, age of onset, time of exposition to antipsychotics and degrees of clinical severity [30,56,57]. In addition, an important limitation of most of these studies is the small sample size used [57], which decreases statistical power and limits more definitive conclusions. Finally, only a few studies have used the VBM approach [39,57]. All these aforementioned factors make data interpretation difficult, reinforcing the need for meta-analytic studies in this area using homogeneous morphometric methodologies and including multiple samples of patients.

A tool for voxel-wise meta-analysis of neuroimaging studies is the anatomic likelihood estimation technique (ALE). ALE incorporates multiple data sets of published coordinates generated by different VBM studies of a given disorder, automatically identifying through a whole-brain activation likelihood map those statistically significant (i.e., the most consistent) brain differences reported across these studies [58-60]. By avoiding the bias inherent in those studies employing manual ROIs and allowing the application of a statistical procedure, by depicting regional brain differences with good spatial resolution and by affording more definitive conclusions than single VBM studies, ALE is considered the most sophisticated and best-validated method of coordinate-based voxel-wise meta-analysis [61,62].

Although a number of well-written and comprehensive literature reviews have been conducted in recent years addressing the role of antipsychotics in the progression of structural brain changes over time in schizophrenia [38,39,41,43], these approaches to date have been only qualitative. No published study to date has used the ALE quantitative approach to conduct meta-analyses of VBM investigations comparing schizophrenia patients on antipsychotics versus unmedicated patients and healthy controls, or comparing schizophrenia patients before and after antipsychotic usage. Considering that some VBM studies have investigated samples comprised of as few as 15 schizophrenia patients, it is timely to apply a meta-analytic approach addressing this question, to quantitatively synthesize the findings of different studies, thus affording greater statistical power through the use of larger samples.

In this study, therefore, we sought to quantitatively review the relevant literature on the association between antipsychotics and structural brain changes in schizophrenia through a meta-analytic approach of VBM studies by using the ALE method. By quantitatively integrating the different foci of structural changes reported in each study, our objective was to establish whether a consistent anatomical pattern across these reported foci can be observed and determine the clusters of significant topographic convergence, ultimately providing a neuroanatomical basis for these changes. We also questioned whether the meta-analytic approach might aid in differentiating between the effects of antipsychotics and those solely related to the disease itself. Alternatively, it might help to organize the data from distinct studies even without further clarifying this dilemma. In this sense, we hypothesized that the quantitative meta-analytic approach, by more consistently identifying the regions affected by antipsychotics, might help to delineate the regional patterns of brain involvement associated with antipsychotic medications and to verify the overlaps with the classical patterns of brain involvement associated with the pathophysiological process during the development of psychosis. The identification of areas of structural brain alterations associated with antipsychotic exposure that differ from those areas commonly associated with the disease would aid in achieving a better understanding of this question.

## Methods

### Data sources and paper selection

We conducted a systematic computerized literature search via the MEDLINE/PubMed (http://www.ncbi.nlm.nih.gov/pubmed), EMBASE (http://www.embase.com), ISI Web of Science (http://newisiknowledge.com/wos), SCOPUS (http://www.scopus.com) and PsycINFO (http://www.apa.org/psycinfo) databases for VBM studies investigating the role of antipsychotic drugs in structural brain changes in samples of patients with schizophrenia. We used the following search keywords in different combinations to generate a list of potentially useful studies: “schizophrenia” (as well as variants, including “psychosis” and “schizoaffective”), crossed with “antipsychotics”, “antipsychotic agents” or “neuroleptics” and neuroimaging stems, including “MRI”, “magnetic resonance imaging”, “VBM” and “voxel-based morphometry”. The search was performed through August 2012, and no restrictions on date of publication or language were applied. We carefully examined all titles and abstracts resulting from these searches to determine which articles met the criteria for inclusion. The full text of all selected articles was evaluated, and the references for each article were also screened to identify additional eligible papers. All studies were obtained from peer-reviewed journals.

**Figure 1:**
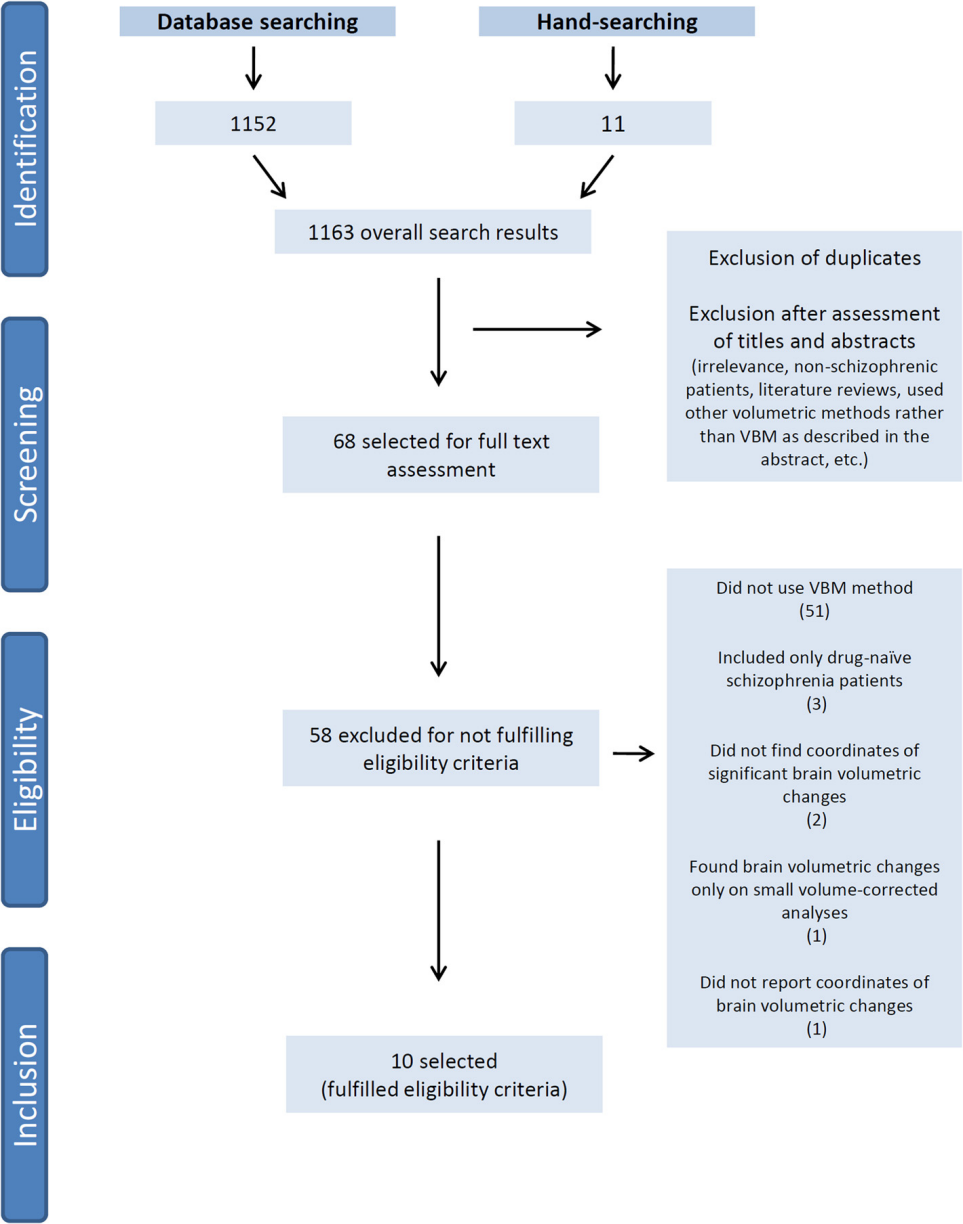
PRISMA flowchart of search results.

We selected studies considering the following inclusion criteria: a) original research articles; b) quantitative automated whole-brain analyses were performed; c) the VBM method was used [26,63]; c) the samples included subjects with schizophrenia using typical or atypical antipsychotics, and comparisons were performed with healthy controls or medication-free subjects, or schizophrenic patients were compared through serial MRI examinations both at baseline and after specific treatment with an antipsychotic; d) the results were normalized to the Talairach [64] or Montreal Neurological Institute (MNI) [65,66] standardized stereotactic spaces; e) after VBM analyses, the peak coordinates of structural brain changes specifically associated with antipsychotics (i.e., after controlling for confounding effects such as the effects of disease, disease duration, etc.) were explicitly reported. In cases where the coordinates were not reported, attempts were made to contact the corresponding author for further details (e-mail and phone contact). The PRISMA (Preferred Reporting Items for Systematic Reviews and Meta-Analyses) flowchart detailing all the steps of the systematic review is provided in Figure 1.

### Meta-analytic techniques

As aforementioned, meta-analysis was carried out by using the ALE method as introduced by Turkeltaub in 2002 [58], with revision by Laird in 2005 [59], Eickhoff in 2009 [60] and Turkeltaub in 2011 [67]; data processing was performed through the GingerALE 2.1 program [59,60,67] (http://www.brainmap.org). The analyses were conducted in Talairach space [64], and, when necessary, we spatially renormalized the MNI coordinates [65,66] published in some studies to Talairach coordinates [64], using the Lancaster’s transform (“icbm2tal”) [68,69].

Briefly, ALE is a method that determines the existence of anatomical convergence among results from different samples and studies, assuming an uncertainty in the location of each reported focus, which should be considered in terms of Gaussian probability density distributions that surround themselves. Therefore, the focus of maximal activation (peak coordinates) represents the center of a three-dimensional Gaussian probability [58]. As recently proposed in the revised ALE algorithm, the use of pre-determined full-width half maximum (FWHM) values for the Gaussian probability distributions in the analyses is no longer required. The values are now empirically determined on the basis of a quantitative uncertainty model whereby the coordinates reported by studies with larger samples are more spatially accurate than those from smaller ones, thus requiring smaller FWHM values [60].

As ALE seeks to determine statistically significant convergence of activation probabilities between experiments, refuting the null hypothesis that the foci are homogeneously distributed throughout the brain [70], the modeled three-dimensional Gaussian probabilities of all foci reported in each experiment are summed in a voxel-wise manner, resulting in modeled activation (MA) maps [60,70]. The voxel-wise union of each computed MA map yields the “true ALE scores”, which demonstrate the convergence of foci through the whole brain across the entire set of studies. Permutation analyses conducted in ALE meta-analyses (using GingerALE, for example) are anatomically unconstrained, including not only the predominant foci within gray matter but also the foci within the deep white matter. Thus, it is a whole-brain analysis that allows conjoint analysis of gray and white matter foci. Finally, to determine the statistical significance, i.e., to assess the validity of convergence found in the true ALE scores over a random convergence (noise), an automated comparison is performed with a computed empirical null distribution of random ALE scores. For this purpose, from each MA map, a voxel is randomly selected and its probability is computed, and the union of these probabilities (as done for the true scores) yields the random score [60].

As described in previous meta-analyses of VBM studies, we adopted a threshold for the map of final ALE scores with a false discovery rate (FDR) corrected at p < 0.05 [71-73] and a cluster extent threshold of 100 mm^3^[72,74]. In addition, we chose the resultant coordinates to be reported for all submaxima in a single ALE cluster (all extrema). Significant clusters were overlaid onto an anatomical Talairach template, Colin1.1.nii (http://www.brainmap.org/ale), using the Mango software (version 2.6, 2012, Research Imaging Institute, University of Texas Health Science Center, USA; http://ric.uthscsa.edu/mango).

## Results

The systematic search yielded 1163 abstracts, of which 68 were initially selected for a full-text screening. One study, by Massana et al. [75], was excluded, as the stereotactic coordinates were not reported in the paper nor provided by the authors after request (personal communication). Another study, by Schaulfelberger et al. [76], was excluded because significant peak coordinates of structural brain changes were found only with small volume correction analysis.

Finally, ten studies [77-86] met the inclusion criteria (PRISMA flowchart in Figure 1). Additional data were necessary for the maps and peak coordinates reported in the study by Molina et al. [86] and were provided by the authors after submitting a request via e-mail (personal communication). Table 1 illustrates the clinical and demographic variables of the subjects included in the ten selected studies, which encompassed a total of 548 individuals (298 patients using typical or atypical antipsychotics and 250 controls). For the effect of reported coordinates included in this meta-analysis, five selected studies were classified as longitudinal [78,79,81,82,84] and five as cross-sectional [77,80,83,85,86]. Table 2 provides details on the methodologies of each selected study.

**Table 1:** Clinical and demographical data from subjects included in the selected studies

**Reference**	**Subjects ( *n *)**						**Gender of patients (F/M)**	**Age of patients (years)**^**a**^	**Education (years)**^**a**^	**Duration of illness**	**Handedness (R/L/A)**
	** Patients**		** Controls**			**Total**					
	**Typical**	**Atypical**	Healthy subjects^*^	**SP**	**MF**						
**Dazzan *et al. *** **2005**[77]	32	30	--	--	22	84	21/41	25 ± 8 to 28.4 ± 7.8	12.3 ± 1.8 to 12.3 ±2.1	19 to 22 weeks^b^	54/8
**Girgis *et al. *** **2006**[78]	--	15	15	15	--	30	8/7	23.6 ± 5.9	13.1 ± 3.2	105.3 ± 94.6 weeks^a^	NA
**Whitford *et al. *** **2006**[79]	--	25	26	25	--	51	10/15	22.1 ± 3.2	NA	5.9 ± 8.2 months^a^	21/4
**Douaud *et al. *** **2007**[80]	--	25	25	--	--	50	7/18	15.9 ± 1.5 to 16.5 ± 1.3	NA	1.4 ± 0.7 year^a^	20/5
**Théberge et al. 2007**[81]	16		16	16	--	32	2/14	25 ± 8	11-13	243 ± 120 weeks^a^	12/3/1
**Stip *et al. *** **2009**[82]	--	15	--	15	--	15	4/11	28.3 ± 9.07	10.6 ± 3.5	5.8 ± 6.2 years^a^	NA
**Tomelleri *et al. *** **2009**[83]	25	45	79	--	--	149	25/45	39.73 ± 10.94	NA	14.13 ± 10.7 years^a^	67/3
**Deng *et al. *** **2009**[84]	6	14	11	--	--	31	11/9	26 ± 10 to 29.9 ± 13.5	NA	NA	18/2
**Chua *et al. *** **2009**[85]	15	5	--	--	25	45	10/10	29 ± 8.6	12 ± 2.9	40.8 ± 50.8 weeks^a^	NA
**Molina *et al. *** **2011**[86]	--	30	31	--	--	61	14/16	34.1 ± 10.6	NA	13.4 ± 5.9 years^a^	NA

*Matched for age and gender to the patients groups; a= mean ± SD; b = median (±SD); NA= data not available; SP = same patients evaluated repeatedly in longitudinal comparisons; MF = medication-free subjects.

**Table 2:** Summary of the methodologies used for each selected study

**Reference**	**Design**	**Methods**	**Stereotactic space**	**Statistical threshold**	**Full-width half-maximum kernel**	**Type of analysis**	**P value**	**Controlling for**
**Dazzan *et al. *** **2005**[77]	Cross-sectional	Comparison of subjects using typical and atypical antipsychotics versus drug-free patients	Talairach	Corr	NA	Whole-brain analysis	P ≤0.002	Age, gender, duration of illness, total symptom scores, length of treatment, premorbid IQ, years of education
**Girgis *et al. *** **2006**[78]	Longitudinal	Comparison between patients using atypical antipsychotics (from baseline to 6-week follow-up) versus healthy controls	Talairach	Unc	12mm	Whole-brain analysis	P ≤0.001	Age, gender, follow-up interval, years of education, socioeconomic status, duration of illness, total symptom scores
**Whitford *et al. *** **2006**[79]	Longitudinal	Comparison between patients using atypical antipsychotics (at first episode psychosis and at 2-3-year follow-up) versus healthy controls	Talairach	Corr	12mm	Whole-brain analysis	P < 0.05	Age, gender, handedness, follow-up interval
**Douaud *et al. *** **2007**[80]	Cross-sectional	Comparison of subjects using atypical antipsychotics versus healthy controls	MNI	Corr	8mm	Whole-brain analysis	P < 0.01	Age, gender, handedness, socioeconomic status
**Théberge et al. 2007**[81]	Longitudinal	Comparison between patients using antipsychotics (at first episode psychosis and at 30-month follow-up) versus healthy controls	Talairach	Corr	12mm	Whole-brain analysis	P <0.001	Age, gender, handedness, parental education
**Stip *et al. *** **2009**[82]	Longitudinal	Comparison between patients using atypical antipsychotics at baseline and at 5.5-month follow-up	MNI	Corr	8mm	Whole-brain analysis	P < 0.01	Non specified
**Tomelleri *et al. *** **2009**[83]	Cross-sectional	Comparison of subjects using atypical and typical antipsychotics versus healthy controls	Talairach	Corr	12mm	Whole-brain analysis	P < 0.01	Gender, duration of illness, total symptom scores
**Deng *et al. *** **2009**[84]	Longitudinal	Comparison between patients using atypical and typical antipsychotics (from baseline to 8-week follow-up) versus healthy controls	MNI	Unc	8mm	Whole-brain analysis	p<0.001	Age, gender, height, handedness
**Chua *et al. *** **2009**[85]	Cross-sectional	Comparison of subjects using atypical and typical antipsychotics versus drug-free patients	Talairach	Corr	4.4mm	Whole-brain analysis	p<0.002	Age, gender, handedness, ethnicity, height, years of education, paternal socio-economic status, total symptom scores
**Molina *et al. *** **2011**[86]	Cross-sectional	Comparison of subjects using atypical antipsychotics versus healthy controls	Talairach	Corr	NA	Whole-brain analysis	p< 0.05	Age, gender, parental socioeconomic status, duration of illness

Corr = corrected for multiple comparisons; Unc = uncorrected for multiple comparisons.

Most of the patients enrolled were using atypical antipsychotics (234; 78.5%), which in these samples included olanzapine, risperidone, quetiapine, sertindole, amisulpiride, clozapine and ziprasidone. From the subset of studies which specifically described the number of patients who had taken each antipsychotic [77,78,80,82-86], olanzapine and risperidone were the most frequently used drugs among these 179 patients treated with atypicals, being used for 40.7% (73) and 30.1% (54) of the patients, respectively. Typical antipsychotics used when considering all the selected studies were chlorpromazine, sulpiride, haloperidol, thioridazine, droperidol, trifluoperazine, zuclopenthixol, fluphenazine and clotiapine. Additional file 1 summarizes the data concerning the antipsychotic drugs used by the patients of these selected studies.

**Figure 2:**
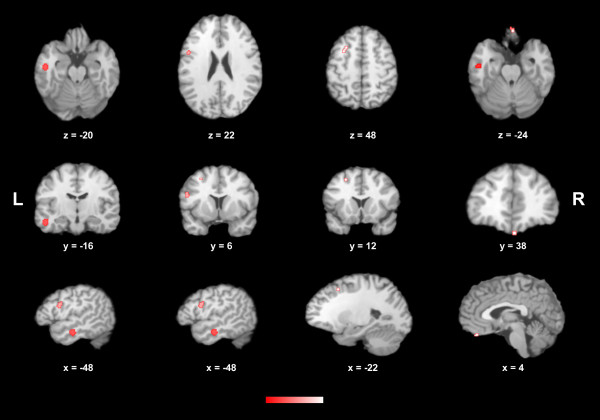
**Areas of statistically significant brain volumetric decreases across the selected studies (displayed in the axial, coronal and sagittal plans) in patients with schizophrenia receiving antipsychotic medications.** Peak coordinates (x;y;z) in the Talairach stereotactic space are presented. *L* left; *R* right.

**Figure 3:**
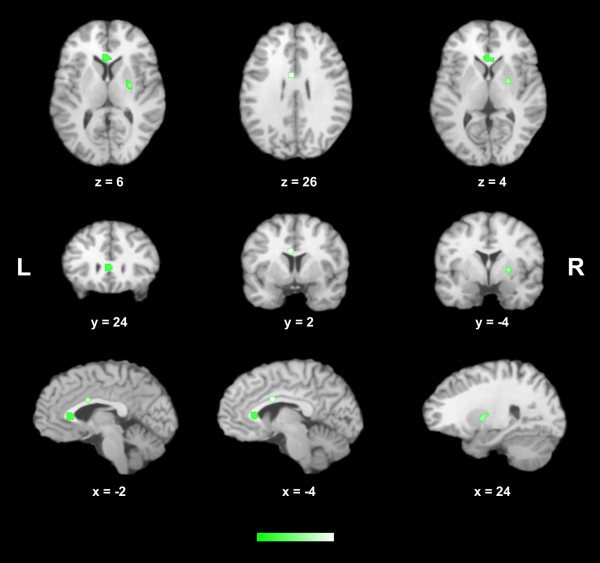
**Areas of statistically significant brain volumetric increases across the selected studies (displayed in the axial, coronal and sagittal plans) in patients with schizophrenia receiving antipsychotic medications.** Peak coordinates (x;y;z) in the Talairach stereotactic space are presented. *L* left; *R* right.

Overall, 105 foci were retrieved in the analysis of the ten studies, 71 of which were related to volumetric gray matter and/or white matter deficits, and 34 to volumetric excesses. Additional file 2 demonstrates in the stereotactic space the foci of reported structural brain changes according to the class of antipsychotics (when available) and the type of alteration in the meta-analyzed studies. As some of the selected studies showed different effects for the use of antipsychotics, i.e., showed areas of both volumetric excesses and volumetric deficits, the meta-analysis was performed separately for those coordinates related to volumetric deficits and for those related to volumetric excesses. Patients using antipsychotic medications had four significant clusters of volumetric deficits in comparison to controls: 1) a cluster of 408 mm^3^ located in the left lateral temporal cortex, BA 20 (peak voxel at Talairach coordinate −48, -16, -20); 2) a cluster of 192 mm^3^ located in the left inferior frontal gyrus, BA 44 (peak voxel at Talairach coordinate −48, 6, 22); 3) a cluster of 120 mm^3^ located in the left superior frontal gyrus, extending to the left middle frontal gyrus, BA 6 (peak voxel at Talairach coordinate −22, 12, 48); and 4) a cluster of 104 mm^3^ located in the right rectal gyrus, BA 11 (peak voxel at Talairach coordinate 4, 38, -24). In addition, patients using antipsychotic medications also had three significant clusters of volumetric excesses in comparison to controls: 1) a cluster of 416 mm^3^ located in the left dorsal anterior cingulate cortex, BA 24 (peak voxel at Talairach coordinate −2, 24, 6); 2) a cluster of 152 mm^3^ located in the left ventral anterior cingulate cortex, BA 24 (peak voxel at Talairach coordinate −4, 2, 26); and 3) a cluster of 264 mm^3^ located in the right putamen (peak voxel at Talairach coordinate 24, -4, 4). The final maps with the resultant significant areas of volumetric deficits and excesses in patients using antipsychotics through the selected studies are displayed in Figures 2 and 3, respectively.

We also conducted sub-analyses comparing the effects of typical and atypical antipsychotics. Three studies did not report peak coordinates according to typicality and were excluded from these sub-analyses [81,84,85]. Volumetric decreases with typicals were found in only one study [77], and thus these foci could not be meta-analyzed. Volumetric increases with typicals were found in two studies [77,83], but no significant clusters were found. Volumetric decreases with atypicals were reported in three studies [79,80,86], retrieving one significant cluster of 456 mm^3^ located in the left temporal lobe, BA 20 (peak voxel at Talairach coordinate −48, -16-, -20). Finally, volumetric increases with atypicals were reported in five studies [77,78,82,83,87], retrieving two significant clusters: 1) a cluster of 160 mm^3^ located in the right putamen (peak voxel at Talairach coordinate 26, -10, 8); and 2) a cluster of 112 mm^3^ located in the left thalamus (peak voxel at Talairach coordinate −2, -26, 4).

## Discussion

Among the several variables that could possibly determine or contribute to the brain structural changes observed in patients with schizophrenia in the numerous neuroimaging studies performed in recent years -- including those specifically related to the illness (age of onset, duration, severity) and the individual (age, gender, scholarity) -- it is the role of antipsychotics that remains a critical question, although possibly still beyond a definitive answer.

A relatively low number of studies have addressed this issue, which is made more difficult by the complex task of harmonizing or balancing the effects of all the other possible variables, and by the crucial necessity of more homogeneous samples of patients, not only with respect to the variables related to the illness and individuals but also to those related to antipsychotics (class, years of exposure, dosages, withdrawals). Indeed, the additional difficulty of recruiting subjects with a psychiatric disease that has a low prevalence rate [88] has led to studies with relatively small numbers of patients. Considering also that the volumetric structural brain alterations in schizophrenia are often very subtle, thus requiring large samples to sufficiently increase the statistical power that would allow for the detection of such alterations [88], it is not surprising that sometimes the results of these distinct studies are somewhat confusing or even contradictory [38,39,41,43].

Whereas studies on the progression of structural brain changes in schizophrenia over time have been consistently replicated through recent years, as previously addressed in the introduction, studies on the role of antipsychotics have been presenting results ranging from brain volumetric reductions [89] to “no effect” [90] to volumetric enlargement [91], for example. Evidence from studies including larger samples of subjects, however, suggests a progressive correlation between brain volumetric reduction and antipsychotic use. In a multi-site longitudinal randomized study carried out with 161 first-episode psychosis patients and 62 healthy controls, Lieberman et al. [92] allocated patients, after a baseline MRI scan and subsequent randomization, to haloperidol or olanzapine groups; they found that patients using haloperidol had a significant grey matter reduction after 12 and 52 weeks in comparison to controls, whereas olanzapine patients did not. Also Cahn et al. [89] prospectively studied 34 first-episode schizophrenia patients (who had taken antipsychotics for up to 16 weeks) and 36 healthy controls who underwent an MRI scan at admission and after 1 year; a higher cumulative dosage of antipsychotics (typicals and atypicals were used in the study) was significantly and independently correlated with total brain volume and gray matter volume reductions, as well as increased volume of lateral ventricles. A longitudinal study by Nakamura et al. [93] (29 first-episode schizophrenia patients, 34 first-episode affective psychosis and 26 healthy controls) showed that schizophrenia patients had smaller gray matter volumes and larger sulcal cerebrospinal fluid and lateral ventricles in comparison to controls. Jayakumar et al. [94] studied 18 antipsychotic-naïve schizophrenia patients and 18 healthy controls; results showed gray-matter volumetric reductions and larger cerebrospinal fluid volumes, as also observed by Davatzikos et al. [95] (32 first-episode neuroleptic-naïve schizophrenia patients, 37 patients treated with antipsychotics and 79 controls). Conversely, some studies on this question have not found significant structural brain changes (volumetric reductions or excesses) when comparing patients and controls. Studies with large samples, for example, such as those by Molina et al. [87] (16 schizophrenia patients and 42 healthy controls), Tauscher-Wisniewski et al. [96] (37 first-episode patients and 37 controls), and Puri et al. [97] (24 first-episode patients and 12 controls), did not identify significant volumetric differences. Recently, Ho et al. [98] performed a large longitudinal MRI study that followed 211 schizophrenia patients over a mean of 7.2 years, aiming to assess four potential variables (illness duration, illness severity, use of antipsychotics and substance abuse) that could have an effect on brain volumes. The authors found that even considering the effects of the other three variables, after statistical controlling, there was still a relationship between the amount of exposure to antipsychotics and brain volumetric reductions. Interestingly, these brain volumetric changes were not solely related to a specific antipsychotic class, but were observed both with typicals and atypicals [98].

The distinct pattern of effects on the brain induced either by typical or atypical drugs was a matter of discussion in some literature reviews, which showed that volumetric brain changes are more related to typicals than atypicals [39,41]. Another review, however, concluded that the literature on structural brain changes and antipsychotic effects is inconsistent (as about half of the longitudinal studies did not find or report progressive brain changes), and the distinct patterns of effects determined by typicals or atypicals were inconsistent as well [38].

To the best of our knowledge, this is the first study to quantitatively assess with a meta-analytic approach the question of structural brain changes and antipsychotic use in schizophrenia by reviewing those studies which directly assessed this question. In 2011, Leung et al. [71] published a meta-analysis of VBM studies using the ALE method to detect antipsychotic-related gray-matter alterations in first-episode schizophrenia patients; however, in contrast to our study, these authors employed an indirect approach, by using subtraction analysis to compare structural brain changes between two separate subsets of meta-analyzed studies, i.e., a subset of studies involving neuroleptic-naïve first-episode schizophrenia patients and another subset of studies involving neuroleptic-treated first-episode schizophrenia patients. Additionally, literature reviews have hitherto been only qualitative, encompassing a large variety of studies carried out through distinct methodologies concerning volume measurement techniques.

The chosen voxel-wise approach using the ALE method confers at least one special advantage over previous reviews, which is the homogeneity of techniques employed by the selected studies regarding volumetry (i.e., the VBM method). Our results revealed a consistent volumetric reduction across the selected studies in the left temporal and frontal lobes, all of them being previously implicated areas reported to be of significance in studies involving patients with schizophrenia and first-episode psychosis [10,25,56,99-104]. Indeed, there is, for example, a body of evidence in the literature associating typical antipsychotics and also risperidone with decreased frontal metabolism in schizophrenia patients [105-108]. In the study by Leung et al. [71], subtraction analysis between the neuroleptic-treated and neuroleptic-naïve groups of first-episode psychosis patients revealed more extensive gray matter volumetric deficits in neuroleptic-treated patients in bilateral insula, medial frontal and inferior frontal gyrus, left parahippocampal gyrus, superior temporal gyrus and right precentral gyrus; of interest, while there were significant gray matter deficits in the frontal lobe of neuroleptic-naïve patients (a finding not related to drug exposure), treated patients also showed still more extensive frontal volumetric reductions, which notes a possible overlapping between the disease-related structural alterations and the effects of antipsychotics.

Another point of interest is the question of volumetric excesses. The finding of volumetric increases in subcortical regions (and in basal ganglia, more specifically) is widely described in the ROI literature and thought to be related to dopaminergic blockade [39] and increased striatal blood flow [109]. Antipsychotic-related increased volumes in the putamen, nucleus caudatus, globus pallidus and thalamus were found in several studies in the literature, both among typicals and atypicals, even after few weeks of treatment [39]. In previously neuroleptic-naïve patients treated with atypicals, however, these basal ganglia alterations are not usually described [39], and atypicals seem to even reverse this effect after patients switch to them [39,43]. In this meta-analysis, the findings of volumetric excesses were observed across studies employing mainly atypical [77,78,82,83,86] but also typical antipsychotics [77,83]. Significant volumetric increases were observed in the cingulate gyrus and putamen, in concordance with some previous findings in the literature. Cumulative exposure to typical antipsychotics, for example, has been associated with a larger cingulate gyrus, an important region for the pathophysiology of schizophrenia [110-112]. McCormick et al. found an increase in anterior cingulate volume with use of typicals after 2–3 years in previously neuroleptic-naïve subjects; increased atypical medication exposure, in contrast, was correlated to decreased anterior cingulate volume [112]. Kopelman et al. also found greater anterior cingulate cortex volumes directly related to typical medication exposure [113]. This follows the same pattern of findings reported in regard to changes in the volume of basal ganglia structures [112], and these similarities may be related to the fact that the anterior cingulate cortex and basal ganglia are structurally and functionally connected, both structures receiving direct dopaminergic innervation from the ventral tegmental area and the substantia nigra [112]. In addition, the reported antipsychotic-induced hypertrophy in putamen, thalamus and caudate nucleus (observed both with typicals and atypicals) may also be related to changes in the synaptic organization of these D2 receptor-rich areas [84,86]. Indeed, the results of our sub-analysis carried out with atypical antipsychotics are in concordance with these findings, indicating significant volumetric increases in the thalamus and putamen.

In considering the results of this meta-analysis, we have to acknowledge a number of limitations. The main limitation concerns the challenge faced by all the selected studies in this field, which is the difficulty of disentangling the effects of drug treatment from those related to the underlying pathology; the results of various studies on this topic allow us currently only to make hypotheses, without reaching definitive conclusions, as is summarized in the last literature reviews [38,39,41,43]. In addition, ethical issues preclude more insightful study designs involving, for example, placebo-treated individuals with schizophrenia and antipsychotic-exposed healthy controls because there is no therapeutic benefit to justify the exposure of a healthy population to the effects of antipsychotics. Thus, we acknowledge that the results currently available in the literature are merely associative and inferential, and they do not necessarily imply causality. Independent of such facts, however, it should be noted that the current study does not intend primarily to question the internal validity of each published work reporting that these changes might be associated to antipsychotics; despite any concerns, these studies point to a possible association, and, methodologically, they represent the best that the current literature has to offer.

Yet, despite the strictness of the eligibility criteria that we applied, which led to a low number of selected studies, other limitations relate to the heterogeneity of search space (variability both in type of antipsychotic examined and in study designs). However, we aimed with this study to establish whether there were clusters of significant topographic convergence of structural brain changes reported in the selected VBM studies, independent of their designs. It is also possible that some factors (such as smoothing kernel, absence of correction for multiple comparisons and cluster size thresholds) that vary across different VBM studies may exert an influence on the final coordinates generated in each study, thus also affecting the results of ALE meta-analyses [71]. It is important to emphasize, however, that the revised ALE algorithm employed in this meta-analysis accounts for the inter-subject and inter-laboratory variability observed in neuroimaging experiments, also employing a null distribution of spatial independence across studies [70].

Due to the limited reported data, we could not control for the effect of potential moderators such as illness duration and illness severity. However, the relatively large overall sample size, combined with strict quality control, yielded a robust meta-analytical approach. Additionally, when meta-analyzing the available foci according to the class of antipsychotics or type of study design in relation to the resultant brain effect (i.e., volumetric excesses, volumetric decreases), excessive fragmentation of the data prevented the findings of significant foci of convergence with typicals. Notably, however, there is currently no clear evidence regarding the effects of typicality on brain regional changes associated with antipsychotics in schizophrenia [38,98].

Other limitations are related to the ALE method, as only those studies reporting peak coordinates can be incorporated into the meta-analysis; thus, those studies that found no significant differences between patients and controls (and consequently did not report peak coordinates) are not included and therefore cannot influence the meta-analysis results. Thus, by considering only studies with positive findings, ALE meta-analyses may carry a bias that overemphasizes the idea of structural brain changes in schizophrenia [71]. In addition, although it is known that the number of included foci affects the analyses using the ALE method, the ideal number of foci for adequate analyses is still undetermined [59].

## Conclusions

In light of the current literature, despite the growing number of studies, the difficulty in reaching more incisive conclusions on this topic is still apparent. However, it seems clear that the idea of antipsychotics as potential agents contributing to the structural brain changes in schizophrenia should certainly be taken into account. These cautious conclusions are necessary based mainly on the fact that the correlations between brain volumes and antipsychotic use reported by some studies do not necessarily imply any causality [98]. Indeed, it remains elusive whether brain structural alterations in schizophrenia are due to the intrinsic pathologic process, antipsychotic use, other variables, or even a combination of all those [42].

Answering these questions is of great clinical importance. If antipsychotics really induce potentially harmful brain volumetric reductions in patients with schizophrenia, then the risks and benefits of these drugs should be carefully considered before prescription; in addition, patients should be appropriately informed about these particular risks and benefits [38]. More consistent answers could be achieved by studies with high statistical power performed longitudinally, homogeneously, with large samples, ideally multi-centric. For now, some practical approaches such as closely assessing the side effects of these drugs for each patient, trying to prescribe the minimal amount that is sufficient to reach the therapeutical objectives, and also considering the inclusion of nonpharmacological treatments [114] seem adequate. For the future, we expect the research to find new drugs with distinct mechanisms of action [114] that could not only induce fewer undesirable effects but could also act on the underlying disease process rather than on just one dimension or symptom.

## Competing interests

The author declares that they have no competing interests.

## Authors’ contributions

UST and GFB contributed to the study design. UST and EPO contributed to the data collection. UST, EPO, SB and GFB interpreted the data and drafted the manuscript. All authors participated in critical revision of the manuscript drafts and approved the final version.

## Pre-publication history

The pre-publication history for this paper can be accessed here:

http://www.biomedcentral.com/1471-244X/13/342/prepub

## Supplementary Material

Additional file 1 Details of the antipsychotic treatment employed among the subjects enrolled in the selected studies. Click here for file

Additional file 2 Foci of reported brain structural changes (cluster center described in the stereotactic space: x,y,z) * according to type of alteration (increase/decrease of gray and white matter) and class of antipsychotics (typicals/atypicals) in the selected studies. Click here for file
