# Supplementary material for: Structural brain changes associated with antipsychotic treatment in schizophrenia as revealed by voxel-based morphometric MRI: an activation likelihood estimation meta-analysis
Source: BMC Psychiatry. 2013 Dec 20;13:342. doi: 10.1186/1471-244X-13-342 (PMC3878502; doi:10.1186/1471-244X-13-342)
Supplement: Additional file 1 — Details of the antipsychotic treatment employed among the subjects enrolled in the selected studies. [file 1471-244X-13-342-S1.docx]

Additional file 1. Details of the antipsychotic treatment employed among the subjects enrolled in the selected studies.

| **Reference**  **Type** | **Dazzan *et al.* 2005 (77)** | | | **Girgis *et al.* 2006 (78)** | | | **Whitford *et al.* 2006 (79)** | | | **Douaud *et al.* 2007 (80)** | | |
| --- | --- | --- | --- | --- | --- | --- | --- | --- | --- | --- | --- | --- |
| **Typicals** | **Drug** | **n** | **Mean dose**  **(mg) *^a^*** | -- | | | -- | | | **--** | | |
|  | Chlorpromazine  Sulpiride  Haloperidol  Thioridazine  Droperidol  Trifluoperazine  Zuclopenthixol | 32 | *269.5 ± 245*  *CPZeq.* |  |  |  |  |  |  |  |  |  |
| **Atypicals** | **Drug** | **n** | **Mean dose**  **(mg)** | **Drug** | **n** | **Mean dose (mg) *^a^*** | **Drug** | **N** | **Mean dose (mg) *^a^*** | **Drug** | **n*** | **Mean dose**  **(mg) *^a^*** |
|  | Olanzapine | 21 | 14 | Risperidone | 15 | 2.67 ± 1.23 | Amisulpride Clozapine Olanzapine Risperidone Quetiapine | 25 | 280 ±272 *CPZeq.* | Olanzapine | 16 | 340 ± 180  *CPZeq.* |
|  | Risperidone | 5 | 4 |  |  |  |  |  |  |  |  |  |
|  |  |  |  |  |  |  |  |  |  | Quetiapine | 3 |  |
|  | Quetiapine | 2 | 400 |  |  |  |  |  |  |  |  |  |
|  |  |  |  |  |  |  |  |  |  | Clozapine | 6 |  |
|  | Sertindole | 1 | 16 |  |  |  |  |  |  | Risperidone | 3 |  |
|  | Amisulpride | 1 | 400 |  |  |  |  |  |  |  |  |  |

Table 2. (Continuation)

| **Reference**  **Type** | **Theberge *et al.* 2007 (81)** | | | **Stip *et al.* 2009 (82)** | | | **Tomelleri *et al.* 2009 (83)** | | |
| --- | --- | --- | --- | --- | --- | --- | --- | --- | --- |
| **Typicals** | **Drug Ω** | **n** | **Mean dose**  **(mg) *^a^*** | -- | | | **Drug** | **N** | **Mean dose**  **(mg) *^a^*** |
|  | Haloperidol | 2 | 154 *±* 150 *CPZeq.* |  |  |  | Haloperidol | 16 | 206.2 ± 186.4  *CPZeq.* |
|  |  |  |  |  |  |  | Chlorpromazine | 3 |  |
|  |  |  |  |  |  |  | Fluphenazine | 2 |  |
|  |  |  |  |  |  |  | Clotiapine | 2 |  |
|  |  |  |  |  |  |  | Zuclopenthixol | 1 |  |
|  |  |  |  |  |  |  | Thioridazine | 1 |  |
| **Atypicals** | **Drug Ω** | **n** | **Mean dose**  **(mg)** | **Drug** | **n** | **Mean dose (mg) *^a^*** | **Drug** | **n** | **Mean dose**  **(mg) *^a^*** |
|  | Olanzapine  Risperidone  Quetiapine  Ziprasidone  Clozapine | 11 | 154 *±* 150 *CPZeq.* | Quetiapine | 15 | 529 ± 138.9 | Olanzapine | 25 | 193.6 ± 92.7  *CPZeq.* |
|  |  |  |  |  |  |  |  |  |  |
|  |  |  |  |  |  |  | Clozapine | 9 |  |
|  |  |  |  |  |  |  |  |  |  |
|  |  |  |  |  |  |  | Risperidone | 9 |  |
|  |  |  |  |  |  |  |  |  |  |
|  |  |  |  |  |  |  | Quetiapine | 2 |  |
|  |  |  |  |  |  |  |  |  |  |

Table 2. (Continuation)

| **Reference**  **Type** | **Deng *et al.* 2009 (84)** | | | **Chua *et al.* 2009 (85)** | | | **Molina *et al.* 2011 (86)** | | |
| --- | --- | --- | --- | --- | --- | --- | --- | --- | --- |
| **Typicals** | **Drug** | **n** | **Mean dose**  **(mg) *^a^*** | **Drug** | **n** | **Mean dose**  **(mg) *^a^*** | -- | | |
|  | Haloperidol | 1 | 100 *CPZeq.* | Haloperidol | 13 | NA |  |  |  |
|  | Flupenthixol | 2 | 100 *CPZeq.* | Trifluoperazine | 1 |  |  |  |  |
|  |  |  |  |  |  |  |  |  |  |
|  | Trifluoperazine | 3 | 160 *CPZeq.* | Sulpiride | 1 |  |  |  |  |
|  |  |  |  |  |  |  |  |  |  |
| **Atypicals** | **Drug** | **N** | **Mean dose (mg) *^a^*** | **Drug** | **N** | **Mean dose (mg) *^a^*** | **Drug** | **n ⱡ** | **Mean dose**  **(mg) *^a^*** |
|  | Amisulpride | 9 | 100 *CPZeq.* | Amisulpride | 5 | NA | Risperidone | 20 | 4.9 ± 2.1 |
|  |  |  |  |  |  |  | Olanzapine | 10 | 9.8 ± 6.21 |
|  | Olanzapine | 1 | 400 *CPZeq.* |  |  |  |  |  |  |
|  |  |  |  |  |  |  | Quetiapine | 6 | 491.7 ± 216.1 |
|  | Risperidone | 2 | 50 *CPZeq.* |  |  |  |  |  |  |
|  |  |  |  |  |  |  | Amisulpride | 4 | 228.3 ± 85.1 |
|  | Quetiapine | 2 | 170 *CPZeq.* |  |  |  | Clozapine | 4 | 237.1 ± 136.3 |
|  |  |  |  |  |  |  |  |  |  |
|  |  |  |  |  |  |  | Ziprasidone | 2 | 122.3 ± 47.7 |

a= mean ± SD; * 2 patients received two or more drugs; CPZeq= chlorpromazine equivalents; ⱡ = 16 patients received more than one drug; Ω = these data are relative to the time of a MRI scan obtained at approximately 30 months after the start of treatment; NA= data not available
