# Supplementary material for: Structural brain changes associated with antipsychotic treatment in schizophrenia as revealed by voxel-based morphometric MRI: an activation likelihood estimation meta-analysis
Source: BMC Psychiatry. 2013 Dec 20;13:342. doi: 10.1186/1471-244X-13-342 (PMC3878502; doi:10.1186/1471-244X-13-342)
Supplement: Additional file 2 — Foci of reported brain structural changes (cluster center described in the stereotactic space: x,y,z) * according to type of alteration (increase/decrease of gray and white matter) and class of antipsychotics (typicals/atypicals) in the selected studies. [file 1471-244X-13-342-S2.docx]

Additional file 2. Foci of reported brain structural changes (cluster center described in the stereotactic space : x,y,z)* according to type of alteration (increase / decrease of gray and white matter) and class of antipsychotics (typicals/atypicals) in the selected studies.

| **Reference**  **Type** | **Dazzan *et al.* 2005 (77)** | **Girgis *et al.* 2006 (78)** | **Whitford *et al.* 2006 (79)** | **Douaud *et al.* 2007 (80)** |
| --- | --- | --- | --- | --- |
| **Typicals** | **Gray matter excesses** | -- | -- | -- |
|  | Right lenticular nucleus  (T = 23.1, -3.1, 2.6) |  |  |  |
|  | **Gray matter deficits** |  |  |  |
|  | Right insula, extending into inferior frontal gyrus, superior temporal gyrus  (T = 37, 12.3, - 4.6)  Left and right paracentral lobules, extending into superior and medial frontal gyri, cingulate gyrus  (T = -0.0, -22.2, 46.7)  Left precuneus  (T = -1.3, -47.4, 49.4) |  |  |  |
| **Atypicals** | **Gray matter excesses** | **Gray matter excesses** | **Gray matter deficits** | **Gray matter deficits** |
|  | Left and right thalamus  (T = -2.9, -25.8, 4.8) | Left superior temporal gyrus  (T = -54, -59, 19)  Left middle temporal gyrus  (T = -57, -54, 14) | Left and right precuneus  (T = -4, -72, 46)  (T = 10, -70, 43)  Left and right subgyral temporal lobe  (T = -49, -17, -21)  (T = 42, -11, -21)  Right superior temporal gyrus  (T = 34, 16, -24)  (T = 38, 6, -26)  (T = 61, -27, 9)  Right parahippocampal gyrus  (T = 16, -46, 8)  Right lingual gyrus  (T = 18, -41, 0)  Right posterior cingulate gyrus  (T = 8, -50, 14)  Left and right cerebellum  (T = -4, -54, -39)  (T = -22, -77, -25)  (T = -24, -75, -33)  (T = -16, -74, -38)  (T = 6, -52, -39)  (T = 10, -54, -33)  Left inferior frontal gyrus  (T = -32, 24, -18)  (T = -40, 26, -18)  (T = -30, 18, -23)  Right postcentral gyrus  (T = 57, -16, 21)  Right inferior parietal lobule  (T = 53, -29, 33) | Left and right Heschl gyrus  (MNI = -48, -18, 10) / (MNI = 50, -22, 8)  Left and right parietal operculum  (MNI = -38, -16, 24) / (MNI = 50, -24, 20)  Left pars opercularis  (MNI = -46, 18, 12) / (MNI = -50, 8, 20)  Left/right supplementary motor area  (MNI = 1, 0, 52)  Left and right post-central gyrus  (MNI = -40, -36, 46) / (MNI = 60, -16, 44)  Right pre-central gyrus  (MNI = 51, -10, 41) / (MNI = 38, 10, 38)  Left frontal eye field  (MNI = -22, 18, 48)  Right anterior cingulate gyrus  (MNI = 14, 8, 34) / (MNI = 14, 40, 30)  (MNI = 10, 22, 25)  Right dorso-lateral prefrontal cortex  (MNI = 20, 44, 20) / (MNI = 20, 44, 32)  Left and right precuneus  (MNI = -12, -61, 49) / (MNI = 24, -70, 32)  Right parieto-occipital fissure  (MNI = 20, -60, 14)  Left calcarine fissure  (MNI = -12, -90, 6)  Left inferior temporal gyrus  (MNI = -50, -16, -22)  Right middle temporal gyrus  (MNI = 58, -11, -16) |
|  |  | **Gray matter deficits** |  |  |
|  |  | Left frontal lobe, rectal gyrus  (T = -2, 43, -26) |  |  |
|  |  | **White matter deficits** |  |  |
|  |  | Left and right cerebrum, subjacent to frontal lobe  (T = -19, 22, 17)  (T = -16, 16, 21)  (T = 23, 19, 18)  Left and right corpus callosum  (T = -10, 23, 15)  (T = 15, 17, 21)  Right anterior cingulum  (T = 7, 28, 15) |  |  |

Table 3. Continuation

| **Reference**  **Type** | **Theberge *et al.* 2007 (81)** | **Stip *et al.* 2009 (82)** | **Tomelleri *et al.* 2009 (83)** |
| --- | --- | --- | --- |
| **Typicals** | **Gray matter deficits** | -- | **Gray matter excesses** |
|  | Left and right frontal lobe  (T = -50, 2, 28)  (T = -18, 64, 18)  (T = 32, 16, 28) |  | Left and right posterior cingulated gyrus  (T = -4, -36, 17)  (T = 6, -36, 17)  Left anterior cingulate gyrus  (T = -4, 2, 26) |
| **Atypicals** |  | **Gray matter excesses** | **Gray matter excesses** |
|  |  | Left and right frontal/inferior orbital  (MNI = -32, 27, -27)  (MNI = 53, 34, -16)  (MNI = 57, 32, -9)  Left and right cingulum anterior  (MNI = -2, 29, 2)  (MNI = -17, 46, -2)  (MNI = 15, 37, 15)  Cingulum anterior  (MNI = 0, 37, 6) | Right thalamus  (T = 18, -17, 5) |

Table 3. Continuation

| **Reference**  **Type** | **Deng *et al.* 2009 (84)** | **Chua *et al.* 2009 (85)** | **Molina *et al.* 2011 (86)** |
| --- | --- | --- | --- |
| **Typicals** | **Gray matter excesses** | **Gray matter excesses** | -- |
|  | Right caudate  (MNI = 17, -2, 18)  Right superior frontal gyrus  (MNI = 11, -6, 73)  (MNI = 67, -21, 44)  Right inferior frontal gyrus  (MNI = 51, 46, 2)  Left and right precentral gyrus  (MNI = -44, 5, 38)  (MNI = 65, 2, 17)  (MNI = 67, -12, 31)  Left superior parietal lobule  (MNI = -29, -57, 46)  Left inferior parietal lobule  (MNI = -32, -44, 44)  Left fusiform gyrus  (MNI = -60, -20, -25)  Right superior occipital gyrus  (MNI = 30, -80, 27)  Right thalamus  (MNI = 8, -13, 14)  Cerebellum left and right posterior lobe  (MNI = -8, -53, -31)  (MNI = 28, -72, -22)  Cerebellum right anterior lobe  (MNI = 12, -52, -6) | Left cingulate gyrus, extending to left medial frontal, left caudate nucleus  (T = -0.6, 21.8, 5.1)  Right cingulate gyrus, extending to right caudate nucleus  (T = 4.9, 21.2, 4) |  |
| **Atypicals** |  |  | **Gray matter excesses** |
|  |  |  | Left and right putamen  (T = -27, -11, 1)  (T = 27,-10, 8 |
|  |  |  | **Gray matter deficits** |
|  |  |  | Left and right anterior cerebellar lobe  (T = -8, -46, -18)  (T = 9, -46, -19)  Left and right insula  (T = -39, -2, 1)  (T = 39, 21, 1)  Left claustrum  (T = -34, -8, 8)  Left and right rectal gyrus  (T = -4, 20, -22)  (T = 4, 37, -23) |
|  | **Gray matter deficits** |  |  |
|  | Left medial frontal gyrus  (MNI = -8, 39, 27)  (MNI = -8, 40, 27)  Left middle frontal gyrus  (MNI = -28, 13, 48)  (MNI = -3, 11, 53)  Cerebellum right posterior lobe  (MNI = 9, -78, -29) |  |  |
|  |  |  | **White matter deficits** |
|  |  |  | Internal and external capsules and parahippocampal  (T = 26, -10, 3)  (T = -2, 27, 0) |

*T = Talairach stereotactic space; MNI = Montreal Neurological Institute stereotactic space.

Pink-colored spaces correspond to those studies which have not described the results of analyses according to antipsychotic type.
